# Differential Regulation of Bladder Pain and Voiding Function by Sensory Afferent Populations Revealed by Selective Optogenetic Activation

**DOI:** 10.3389/fnint.2018.00005

**Published:** 2018-02-12

**Authors:** Jennifer J. DeBerry, Vijay K. Samineni, Bryan A. Copits, Christopher J. Sullivan, Sherri K. Vogt, Kathryn M. Albers, Brian M. Davis, Robert W. Gereau

**Affiliations:** ^1^Department of Anesthesiology and Perioperative Medicine, University of Alabama at Birmingham, Birmingham, AL, United States; ^2^Department of Anesthesiology, Washington University Pain Center, St. Louis, MO, United States; ^3^Department of Neurobiology, Center for Neuroscience at the University of Pittsburgh, Pittsburgh, PA, United States; ^4^Pittsburgh Center for Pain Research, Center for Neuroscience at the University of Pittsburgh, Pittsburgh, PA, United States

**Keywords:** micturition, voiding, interstitial cystitis, TRPV1, Na**_v_**1.8, visceral, optogenetics, channelrhodopsin

## Abstract

Bladder-innervating primary sensory neurons mediate reflex-driven bladder function under normal conditions, and contribute to debilitating bladder pain and/or overactivity in pathological states. The goal of this study was to examine the respective roles of defined subtypes of afferent neurons in bladder sensation and function *in vivo* via direct optogenetic activation. To accomplish this goal, we generated transgenic lines that express a Channelrhodopsin-2-eYFP fusion protein (ChR2-eYFP) in two distinct populations of sensory neurons: TRPV1-lineage neurons (*Trpv1*^Cre^;Ai32, the majority of nociceptors) and Na_v_1.8^+^ neurons (*Scn10a*^Cre^;Ai32, nociceptors and some mechanosensitive fibers). In spinal cord, eYFP+ fibers in *Trpv1*^Cre^;Ai32 mice were observed predominantly in dorsal horn (DH) laminae I-II, while in *Scn10a*^Cre^;Ai32 mice they extended throughout the DH, including a dense projection to lamina X. Fiber density correlated with number of retrogradely-labeled eYFP+ dorsal root ganglion neurons (82.2% *Scn10a*^Cre^;Ai32 vs. 62% *Trpv1*^Cre^;Ai32) and degree of DH excitatory synaptic transmission. Photostimulation of peripheral afferent terminals significantly increased visceromotor responses to noxious bladder distension (30–50 mmHg) in both transgenic lines, and to non-noxious distension (20 mmHg) in *Scn10a*^Cre^;Ai32 mice. Depolarization of ChR2+ afferents in *Scn10a*^Cre^;Ai32 mice produced low- and high-amplitude bladder contractions respectively in 53% and 27% of stimulation trials, and frequency of high-amplitude contractions increased to 60% after engagement of low threshold (LT) mechanoreceptors by bladder filling. In *Trpv1*^Cre^;Ai32 mice, low-amplitude contractions occurred in 27% of trials before bladder filling, which was pre-requisite for light-evoked high-amplitude contractions (observed in 53.3% of trials). Potential explanations for these observations include physiological differences in the thresholds of stimulated fibers and their connectivity to spinal circuits.

## Introduction

The urinary bladder is innervated by primary sensory neurons with somata in the dorsal root ganglia (DRG) giving rise to lightly-myelinated (Aδ) or unmyelinated (C) axons, the majority of which are polymodal mechanosensors (Sengupta and Gebhart, 1994a; Su et al., 1997; Shea et al., 2000). Polymodal mechanosensitive bladder afferents fall into low threshold (LT) and high threshold (HT) categories, distinguishable by their response to luminal distension (Sengupta and Gebhart, 1994a,b). In contrast to cutaneous sensory neurons, response threshold and fiber type of visceral sensory neurons do not correlate (Sengupta and Gebhart, 1994a; Su et al., 1997; Shea et al., 2000). Visceral LT afferents have extrapolated thresholds that are less than one-tenth the threshold of HT afferents, but LT afferents can code noxious distension pressures by increasing firing rate up to twice the frequency seen at threshold. Thus, both LT and HT visceral sensory neurons may be defined as nociceptors.

Pathological changes in bladder sensation often manifest as increased urinary frequency, with pain or without pain, as seen in interstitial cystitis (IC) and overactive bladder (OAB), respectively. Efficacious treatment strategies for stratified patient groups require an understanding of the physiological, molecular, and anatomical sensory components that differentiate bladder function and pain. Animal studies of bladder sensation that have utilized neurotoxins to desensitize C-fiber afferents suggest that mechanosensitive Aδ-fiber bladder afferents are responsive to physiologic changes in intravesical pressure (IVP) during normal bladder filling, while polymodal C-fiber afferents are “recruited” during noxious stimulation or tissue injury (Mallory et al., 1989; Cheng et al., 1993, 1995). However, other studies have shown both loss- and gain-of-function bladder phenotypes in mice following antagonism or knockout of transient receptor potential cation channel subfamily V, member 1 (TRPV1; Birder et al., 2002; Daly et al., 2007; Charrua et al., 2009; Yoshiyama et al., 2015), a non-specific cation channel that is preferentially expressed on peptidergic C-fiber afferents (Michael and Priestley, 1999; Leffler et al., 2006; Cavanaugh et al., 2011a). Additional studies that have manipulated the expression or function of Na_v_1.8, a TTX-resistant voltage-gated sodium channel expressed by a combination of myelinated and unmyelinated afferents including putative C-low-threshold mechanoreceptors (CLTMs; Shields et al., 2012) have demonstrated changes in both normal and pathological bladder sensation (Yoshimura et al., 2001; Laird et al., 2002). It is unclear whether changes in bladder sensation observed in these studies are due to the loss of specific channel activity vs. altered function in the afferent populations in which they are expressed.

Optogenetics combines genetic and optical technologies to directly excite or inhibit genetically defined cells with spatial and temporal precision (Boyden et al., 2005). The excitatory opsin, channelrhodopsin-2 (ChR2), is a blue light (~470 nm)-sensitive ion channel, opening of which causes transducer-independent depolarization and action potential firing in ChR2-expressing neurons. Recent studies demonstrate that activation of ChR2-expressing sensory neurons can evoke nocifensive behaviors in mice (Daou et al., 2013; Boada et al., 2014; Iyer et al., 2014; Baumbauer et al., 2015; Montgomery et al., 2015; Park et al., 2015; Copits et al., 2016; Samineni et al., 2017b). The aim of the current study was to use an *in vivo*, direct method of neuronal stimulation, unbiased by dependence on mechanical or chemical transduction, to determine whether distinct components of bladder sensation that relate to nociception vs. micturition are intrinsically contributed by defined populations of bladder afferent neurons. To achieve this, we utilized a Cre-lox recombination approach to express Channelrhodopsin-2-eYFP fusion protein (ChR2-eYFP) in lineage *Trpv1*- and *Scn10a*-expressing sensory neurons. We found that optical stimulation of the peripheral terminals of ChR2-expressing bladder primary sensory neurons increased nociceptive reflex behavior to mechanical distension of the bladder in both *Trpv1*^Cre^;Ai32 and *Scn10a*^Cre^;Ai32 mice. However, optical stimulation of the two populations had differential effects on cystometric measures of bladder function.

## Materials and Methods

### Animals

*Trpv1*^Cre^;R26-LSL-ChR2-eYFP and *Scn10a*^Cre^;R26-LSL-ChR2-eYFP mice were bred by crossing female Ai32 mice (The Jackson Laboratory, stock #024109) heterozygous or homozygous for the Rosa-CAG-LSL-ChR2(H134R)-eYFP-WPRE conditional allele with a *loxP*-flanked STOP cassette preventing transcription of the ChR2(H134R)-eYFP fusion gene (Madisen et al., 2012) with male Cre recombinase-expressing BAC transgenic mice. *Trpv1*^Cre^ mice (The Jackson Laboratory, stock #017769) were used to target ChR2-eYFP expression to lineage *Trpv1*+ neurons, comprising virtually all C-fiber afferents (Cavanaugh et al., 2009, 2011b; Mishra et al., 2011). SNS^Cre^ mice that express Cre recombinase under the regulatory elements of the *Scn10a* gene (Agarwal et al., 2004), which encodes the TTX-resistant Na1.8 sodium channel, were used to target ChR2-eYFP to a combination of C- and Aδ-fiber afferents (Shields et al., 2012). It should be noted that in SNS^Cre^ (referred to hereafter as *Scn10a*^Cre^) mice, expression of Cre is observed in all Na_v_1.8+ neurons, but not all Cre-expressing cells express Na_v_1.8 in adult mice (Liu et al., 2010; Shields et al., 2012; Chiu et al., 2014). As such, expression of ChR2 in adult mice is not Na_v_1.8 or Trpv1 “specific”. We recently reported a characterization of the expression pattern of ChR2-eYFP in sensory neurons in the same mouse lines used here to generate ChR2 expression driven by the *Scn10a*^Cre^ and *Trpv1*^Cre^ mice (Park et al., 2015). Female offspring harboring a *cre* transgene and that were heterozygous for the Rosa-CAG-LSL-ChR2(H134R)-eYFP-WPRE allele were studied. Experiments were restricted to females due to difficulty in transurethral bladder cannulation without invasive surgical incision in males.

### Retrograde Labeling and Histology

The number of retrogradely labeled L6 DRG neurons expressing the ChR2-eYFP fusion protein was determined in *Trpv1*^Cre^;Ai32 and *Scn10a*^Cre^;Ai32 mice. Mice (*n* = 3–4 per group) were anesthetized with isoflurane and the bladder was isolated via a midline laparotomy under aseptic conditions. Three to five 5 μl injections of AlexaFluor 555 conjugated to the beta subunit of cholera toxin (CTβ-555; Molecular Probes) were made into the bladder wall. Prior to injection, the needle was tunneled subserosally to prevent back flow from the injection site upon needle withdrawal, during which injection sites were swabbed. Abdominal incisions were sutured and mice recovered for 4–5 days. We have previously applied CTβ onto the serosal surface of pelvic viscera and adjacent tissues and found an average of two CTβ-positive cells in L6 ganglia (Christianson et al., 2006). For tissue harvest, mice were deeply anesthetized and perfused with 4% paraformaldehyde. L6 DRG were dissected, cryoprotected in 30% sucrose, and sectioned (16 μm) on a cryostat. Z-stacks were collected for each section at 20× and a maximum intensity projection was obtained using a Nikon A1R upright resonant scanning confocal microscope and Nikon Elements software. Images of Z-stacks were viewed in Adobe Photoshop employing the “Channels” feature to separate color components to determine which cells expressed eYFP and/or were retrogradely labeled by CTβ-555. Using images of individual channels, cells with signals ≥5 standard deviations above background fluorescence were considered positive and counted. At least three sections separated by a minimum of 50 μm were analyzed in a blinded fashion for each ganglion.

### Photostimulation

Optical stimulation was performed using a 473 nm, 150 mW diode-pumped solid-state (DPSS) laser. In visceromotor reflex (VMR) studies, a fiber optic (200 μm diameter core; BFH48-200-Multimode, NA 0.48; Thorlabs) was coupled to the laser and connected to the transurethral catheter via a Y-shaped connector. The fiber tip was positioned 0.1 mm beyond the tip of the catheter in the bladder lumen. Photostimulation was 10 mW/mm^2^ maximal intensity except where noted for stimulation intensity-response curves. For cystometric studies, photostimulation was delivered transabdominally at 50 mW/mm^2^ maximal intensity.

### Dissociated Neuron Electrophysiology

DRG were dissected from Trpv1^Cre^;Ai32 and Scn10a^Cre^;Ai32 mice in ice-cold Ca^2+^/Mg^2+^-free Hank’s buffered saline solution (HBSS) containing 10 mM HEPES. The tissue was digested with 45 U papain (Worthington Biochemical) in HBSS+HEPES for 20 min at 37°C, washed three times with 3 ml of HBSS+HEPES at 37°C and digested in collagenase (1.5 mg/ml; Sigma) for an additional 20 min at 37°C. DRG were rinsed with HBSS+HEPES and mechanically dissociated by gentle trituration in Neurobasal A medium (Gibco) containing 5% fetal bovine serum (Life Technologies), 2 mM GlutaMAX (Life Technologies), 1×B27 supplement (Gibco), and 100 U/ml penicillin/streptomycin (Life Technologies). The DRG suspension was filtered using a 40 μm nylon filter, centrifuged (1000 *g*) for 3 min, resuspended and then centrifuged (1000 *g*) an additional 3 min. Cells were resuspended in medium and seeded on glass coverslips pre-coated with collagen and poly-D-lysine (Sigma). Cells were incubated at 37°C with 5% CO_2_ for 72 h before electrophysiological recordings.

### Spinal Cord Slice Electrophysiology

Transverse spinal cord slices were prepared from 6 week to 8 week old *Trpv1*^Cre^;Ai32 and *Scn10a*^Cre^;Ai32 mice using a protective cutting method (Ting et al., 2014). Animals were deeply anesthetized with ketamine/xylazine and transcardially perfused with room temperature NMDG-substituted aCSF containing (in mM): 93 N-methyl-D-glucamine, 2.5 KCl, 1.25 NaH_2_PO_4_, 30 NaHCO_3_, 20 HEPES, 25 glucose, 5 ascorbic acid, 2 thiourea, 3 sodium pyruvate, 12 N-acetyl-L-cysteine, 0.5 CaCl_2_, 10 MgSO_4_ (pH to 7.3 with HCl; 300–305 mOsm). A ventral laminectomy was performed to extract the spinal cord, and nerve roots and overlying dura mater were carefully removed. Lumbosacral (LS) segments were embedded in 2% low-melting agarose (Sigma; A6013) dissolved in NMDG solution. 350 μm thick slices were cut using a Vibratome VT1000s (Leica) at room temperature, and transferred to an oxygenated recovery chamber containing NMDG-aCSF for 10–12 min at 32–34°C. Slices were then moved to a holding chamber containing oxygenated aCSF, comprised of (in mM): 124 NaCl, 2.5 KCl, 1.25 NaH_2_PO_4_, 24 NaHCO_3_, 5 HEPES, 12.5 glucose, 2 CaCl_2_, 1 MgCl_2_ (pH = 7.3, 300–305 mOsm) and kept for up to 8 h. Slices were transferred to the chamber of an upright Olympus BX-51 microscope and perfused continuously with oxygenated room temperature aCSF at ~2 ml/min. Spinal cord lamina were identified with a 4× objective, and the laminar position of all neurons was confirmed after recordings. Dorsal horn (DH) neurons were visualized through a 40× objective using IR-DIC microscopy. Whole-cell patch-clamp recordings were performed using thick-walled borosilicate glass pipettes with resistance values of 3–5 MΩ, when filled with (in mM): 105 CsMeSO_3_, 15 CsCl, 8 NaCl, 0.2 EGTA, 10 HEPES, 4 MgATP, 0.4 Na_2_GTP, 10 sodium phosphocreatine, 10 tetraethylamine chloride, 3 QX314 chloride (pH = 7.3 with CsOH, 291 mOsm). Recordings were made using Patchmaster software controlling a HEKA EPC10 amplifier. After gigaseal formation and stable whole-cell access, EPSCs were elicited from ChR2-expressing primary afferent fibers with 1 ms pulses of 470 nm blue light (Thorlabs; M470L2) delivered at 0.05 Hz. Whole field illumination was achieved using a custom-made LED set-up with Köhler illumination, coupled to the back fluorescent port of the microscope. Light intensity was calibrated to be 10 mW/mm^2^ at the surface of the slice using a photodiode (Thorlabs; S120C) and power meter (Thorlabs; PM100D). Neurons were voltage clamped at −70 mV and series resistance was compensated at 60% for all recordings. Series resistance values were <30 MΩ, and recordings were discarded if they changed by more than 20%.

### Visceromotor Reflex Behavior

The VMR, quantified using abdominal electromyograph (EMG) responses, is a reliable behavioral index of visceral nociception in rodents (Ness et al., 1991, 2001; Castroman and Ness, 2001; Crock et al., 2012; DeBerry et al., 2015). EMG responses to phasic urinary bladder distension (UBD) were recorded in lightly anesthetized, *Trpv1*^Cre^;Ai32 and *Scn10a*^Cre^;Ai32 mice. Mice were anesthetized with inhaled isoflurane (2% in oxygen) and silver wire electrodes were placed in the oblique abdominal muscle and subcutaneously across the abdominal wall (as a ground) to allow differential amplification of abdominal EMG signals. A lubricated, 24-gauge angiocatheter was passed into the bladder via the urethra for UBD. After surgical preparation, isoflurane was reduced to ~1% until a flexion reflex response was present (evoked by pinching the paw), but spontaneous escape behavior and righting reflex were absent. After the preferred level of anesthesia was attained, no adjustments were made to the isoflurane for the length of the experiment. Mice were not restrained in any fashion and body temperature was monitored and maintained at 37°C throughout the experiment using an overhead radiant light. UBD consisted of compressed air delivered via the transurethral catheter using a custom-made, automated distension control device (Washington University School of Medicine Electronic Shop). Phasic UBD consisted of graded distensions at pressures of 20–50 mmHg (20 s duration; 3× each pressure, 5 min inter-trial interval). EMG signals were relayed in real time using a Grass P511 preamplifier (Grass Technologies) to a PC via a WinDaq DI-720 module (Dataq Instruments), and data were exported to Igor Pro 6.05 software (Wavemetrics). Baseline EMG activity was subtracted from EMG during UBD, rectified, and integrated to obtain distension-evoked EMG responses. Values reported represent the average of the three trials for each pressure. Distension-evoked EMG is presented as area under the curve.

### Cystometry

Cystometrograms (CMGs) were performed in *Trpv1*^Cre^;Ai32 and *Scn10a*^Cre^;Ai32 mice under urethane anesthesia (1.25 g/kg, i.p.). A 24-gauge angiocathether was passed into the bladder lumen through the urethra for saline infusion and measurement of IVP via an in-line, low volume pressure transducer. A midline abdominal incision was made through the skin and rectus muscle and the tissues were gently retracted. The urinary bladder was isolated and periodically irrigated throughout the experiment. Approximately 20 min following urethane administration, saline infusion was initiated at a rate of 20 μL/min to elicit phasic detrusor contractions during bladder filling. After 15 min of recording (0.3 ml total volume), saline infusion was stopped, the catheter was disconnected, and the bladder was emptied using manual compression of the lower abdomen (Credé’s maneuver). The catheter was reconnected to the infusion pump and transducer. Bladder contractile responses were then quantified in response to photostimulation (1, 2 and 5 s of the empty bladder, and then again after saline was infused (20 μL/min) into the bladder to 80% of the micturition threshold (MT), defined as the intravesical volume immediately preceding the onset of the first contractile response of magnitude ≥7.5 mmHg (Cockayne et al., 2000). Photostimulation was restricted to a 3 mm^2^ area of bladder tissue using an opaque shield. Body temperature was maintained at 37°C throughout surgical preparation and data collection using a heating pad placed underneath the animal. IVP was recorded continuously (Spike2 software, Cambridge Electrical Design). Baseline pressure was calculated as the average IVP over a 1 min period prior to saline infusion. Pressure threshold (PT) was defined as the IVP at the MT. Light stimulation-evoked ΔIVP was calculated as the peak IVP after stimulus minus the IVP at stimulus onset.

### Statistical Analyses

Data are presented as mean ± SEM except where noted. Retrogradely labeled eYFP+ DRG neuron counts were compared by a Chi-square test and a Kolmogorov-Smirnov test was used to compare cumulative distributions of cell size. The VMR, quantified as abdominal EMG responses to graded UBD with and without concurrent photostimulation, was compared using a two-way ANOVA with repeated measures and Bonferroni post-tests. Baseline IVP, MT and PT were compared using *t*-tests. Differences in the presence of laser-evoked bladder contractile activity were determined using Chi-square tests. Two-way ANOVAs were used to analyze the effects of stimulus duration on changes in IVP during empty and saline trials. EPSC amplitude and charge transfer during spinal cord recordings were compared with unpaired *t*-tests. A *P*-value of <0.05 was considered significant for all statistical comparisons.

## Results

### Peripheral and Central Projections of eYFP+ Fibers Are Differentially Distributed in *Trpv1*^Cre^;Ai32 and *Scn10a*^Cre^;Ai32 Mice

**Figure 1:**
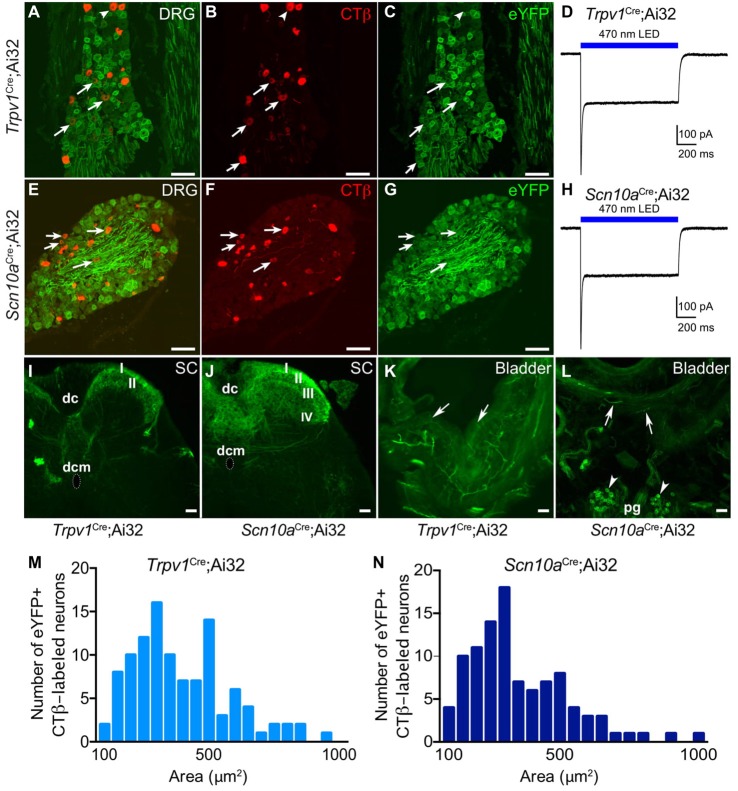
Histological characterization of eYFP+ neurons in *Trpv1*^Cre^;Ai32 and *Scn10a*^Cre^;Ai32 mice. Histological characterization of L6-S1 dorsal root ganglia (DRG) from *Trpv1*^Cre^;Ai32 **(A–C)** and *Scn10a*^Cre^;Ai32 **(E–G)** mice confirms eYFP reporter expression in bladder projecting CTβ+ DRG neurons. Arrows indicate neurons that co-label for CTβ and eYFP. Voltage clamp recordings from isolated *Trpv1*^Cre^;Ai32** (D)** and *Scn10a*^Cre^;Ai32 **(H)** ChR2+ DRG neurons show 1 s blue light stimulation resulted in inward current. **(I)** In *Trpv1*^Cre^;Ai32 mice, eYFP+ terminals were observed in the most superficial layers of the dorsal horn (DH; laminae I-II), and to a lesser degree in lamina X (dorsal commissure; dcm) with sparse projections to the sacral parasympathetic nucleus. dc, dorsal columns. **(J)** In *Scn10a*^Cre^;Ai32 mice, dense eYFP+ afferent terminal distribution is observed in laminae I-V and X, with very few terminals in the sacral parasympathetic nucleus. **(K,L)** In bladder, tissue, eYFP+ fibers were seen in both mouse lines branching from large nerves at the base of the bladder and the outer muscular layers, coursing toward the lumen. Fibers often terminated in the urothelium in *Trpv1*^Cre^;Ai32 mice. In *Scn10a*^Cre^;Ai32 mice, the parasympathetic postganglionic neurons (pg) at the base of the bladder also expressed eYFP (arrowheads). **(M,N)** Cumulative distributions of cell area for eYFP+ CTβ-labeled neurons in *Trpv1*^Cre^;Ai32 and *Scn10a*^Cre^;Ai32 mice did not statistically differ (Kilmogorov-Smirnov test, *P* = 0.97, *n* = 107 for *Trpv1*^Cre^;Ai32 and *n* = 100 for *Scn10a*^Cre^;Ai32). Scale bars represent 100 μm for **(A–G)** and 50 μm for **(I–L)**.

Histological characterization of retrogradely labeled CTβ+ neurons revealed numerous eYFP-expressing neurons projecting to the bladder from LS DRG in *Trpv1*^Cre^;Ai32 (Figures 1A–C) and *Scn10a*^Cre^;Ai32 (Figures 1E–G) mice. However, there were significantly more eYFP+ bladder neurons in *Scn10a*^Cre^;Ai32 mice than in *Trpv1*^Cre^;Ai32 mice (100/122 vs. 107/172, respectively; Chi-square, *P* < 0.001; *n* = 3–4/group). Cell area distributions (Figures 1M,N) show a wide range of labeled neurons. CTβ+ eYFP-expressing neurons ranged from 79.60 to 953.12 μm^2^ (mean = 314.41 μm^2^, sd = 177.21 μm^2^) in *Scn10a*^Cre^;Ai32 mice and from 79.48 to 935.0 μm^2^ (mean = 367.18 μm^2^, sd = 184.66 μm^2^) in *Trpv1*^Cre^;Ai32 mice with no significant difference between cumulative distributions (Kolmogorov-Smirnov test, *P* = 0.97).

ChR2+ DRG neurons from *Trpv1*^Cre^;Ai32 and *Scn10a*^Cre^;Ai32 mice exhibited inward currents upon blue light illumination in voltage clamp recordings (Figures 1D,H), in accordance with our previous observations (Park et al., 2015). Examination of the LS spinal cord showed that eYFP-expressing fibers project to superficial and deep laminae of the spinal DH, including all regions previously shown to receive projections from bladder afferents (Nadelhaft et al., 1992; Birder and de Groat, 1993), but we observed differences in terminal density in different spinal laminae. In *Trpv1*^Cre^;Ai32 mice (Figure 1I), the heaviest distribution of eYFP+ terminals was in the most superficial layers of the DH (laminae I-II) and to a lesser degree in lamina X (dorsal commissure) with sparse projections to parasympathetic preganglionic neurons in the sacral parasympathetic nucleus (SPN; although recent articles argue that these cells have more in common with neurons in the sympathetic intermediolateral cell column (IML) found at T1-L2 spinal cord levels (Espinosa-Medina et al., 2016, 2017)). In contrast, *Scn10a*^Cre^;Ai32 mice exhibited dense afferent terminal distribution to laminae I–V and X, with very few terminals observed in the SPN (Figure 1J).

In bladder, eYFP+ fibers were seen in both *Trpv1*^Cre^;Ai32 (Figure 1K) and *Scn10a*^Cre^;Ai32 (Figure 1L) mice, branching from large nerves at the base of the bladder and the outer muscular layers, coursing toward the lumen, and often terminating in the urothelium. In *Scn10a*^Cre^;Ai32 mice, the parasympathetic postganglionic neurons found at the base of the bladder also expressed eYFP (Figure 1L).

### Optogenetic Stimulation of Defined Subpopulations of Bladder Afferent Terminals Modulates Bladder Nociception and Voiding

**Figure 2:**
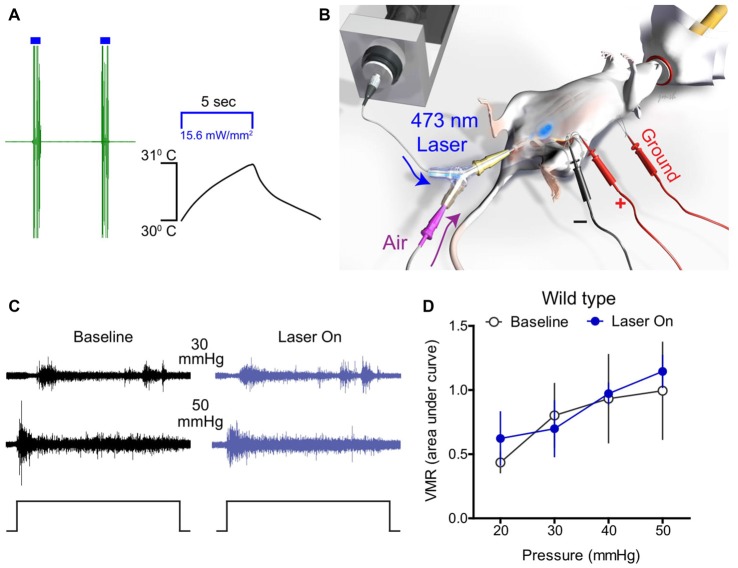
Optical stimulation of bladder afferents did not change bladder distension-evoked responses in wild type mice.** (A)** A representative trace shows that optical stimulation of glabrous skin elicited a robust hind paw withdrawal-evoked abdominal electromyograph (EMG) response in ChR2-expressing mice. Thermistor measurements of skin temperature at the site of stimulation skin show that temperature did not increase more than 1°C upon laser illumination, suggesting that increased EMG activity was not a heat-evoked response. **(B)** Schematic depicting bladder distension, visceromotor reflex (VMR) recording, and optical stimulation setup. **(C)** Representative raw EMG traces from wild type mice during 30 and 50 mmHg bladder distension before (baseline) and during (laser on) blue light stimulation. **(D)** Transurethral fiber optic delivery of blue light to the bladder lumen (laser on) did not affect the evoked response to bladder distension compared with baseline (pre-laser) responses in wild type mice (*P* > 0.05, two-way ANOVA; *n* = 8 mice).

**Figure 3:**
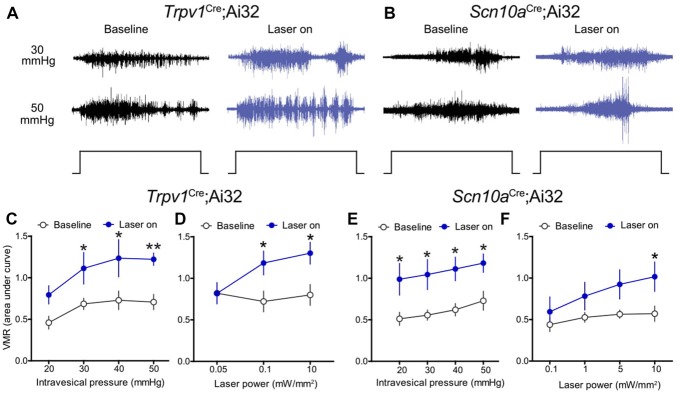
Optical stimulation of ChR2+ bladder afferents potentiated bladder nociception.** (A,B)** Representative raw EMG traces from *Trpv1*^Cre^;Ai32 and *Scn10a*^Cre^;Ai32 mice during 30 and 50 mmHg bladder distension before (baseline) and during (laser on) blue light illumination. **(C)** Transurethral fiber optic delivery of blue light to the bladder lumen (laser on) in *Trpv1*^Cre^;Ai32 mice significantly increased the evoked responses to bladder distension compared with pre-laser (baseline) responses (**P < 0.01, two-way ANOVA; *n* = 8 mice). Significant potentiation occurred at noxious distension pressures (30–50 mmHg, all *P* values <0.05). **(D)** Potentiation of the VMR was light intensity-dependent in *Trpv1*^Cre^;Ai32 mice (*P < 0.05, two-way ANOVA; *n* = 6 mice). **(E)** Optical stimulation of ChR2+ bladder afferents (laser on) significantly increased the evoked response to bladder distension compared with pre-laser (baseline) responses in *Scn10a*^Cre^;Ai32 mice (*P < 0.05, two-way ANOVA; *n* = 8 mice). Distension-evoked responses were potentiated at both noxious (30–50 mmHg, *P values <0.05) and non-noxious (20 mmHg, *P < 0.05) pressures. **(F)** As in *Trpv1*^Cre^;Ai32 mice, potentiation of the VMR was light intensity-dependent in *Scn10a*^Cre^;Ai32 mice (*P < 0.05, two-way ANOVA; *n* = 6 mice).

Initially, optical stimulation was applied to the hind paw of lightly anesthetized ChR2-expressing mice to assess nocifensive responses. Light stimulation of the glabrous skin elicited a robust hind paw withdrawal-evoked abdominal EMG response (Figure 2A), consistent with previously published studies (Ji et al., 2012; Baumbauer et al., 2015). Thermistor measurements of skin temperature at the site of stimulation suggest that this was not a heat-evoked response, since skin temperature did not increase more than 1°C upon exposure to the laser for 5 s (Figure 2A). To study whether optogenetic stimulation of peripheral bladder afferent terminals in *Trpv1*^Cre^;Ai32 and *Scn10a*^Cre^;Ai32 mice could evoke or modulate nociceptive reflex behaviors, VMR recordings during graded UBD in conjunction with transurethral laser stimulation were performed (Figure 2B). Graded UBD produced pressure-dependent increases in VMRs in wild type mice that were not altered by laser stimulation (*n* = 8; two-way ANOVA, *F*_(1,42)_ = 0.10, *P* > 0.05; Figures 2C,D). In contrast, concurrent transurethral laser stimulation significantly increased UBD-evoked VMRs in both *Trpv1*^Cre^;Ai32 mice (*n* = 8; two-way ANOVA, *F*_(1,42)_ = 17.71, *P* < 0.01; Figures 3A,C) and *Scn10a*^Cre^;Ai32 mice (*n* = 8; two-way ANOVA, *F*_(1,42)_ = 8.76, *P* < 0.01; Figures 3B,E). Interestingly, laser stimulation significantly increased VMRs in both groups during noxious distension (30–50 mmHg; all *P* values <0.05), but only in *Scn10a*^Cre^;Ai32 mice during non-noxious distension (20 mmHg; *P* < 0.05). Laser power of ≥0.1 mW/mm^2^ (Figure 3D) and ≥5 mW/mm^2^ (Figure 3F), respectively, effectively potentiated UBD-evoked VMRs in *Trpv1*^Cre^;Ai32 and *Scn10a*^Cre^;Ai32 mice. VMR behavior was not observed in response to transurethral laser stimulation alone, but could be evoked in both groups via transabdominal, direct (without the use of a fiber optic) photostimulation of the bladder at 50 mW/mm^2^ maximal intensity (data not shown). In the latter case, the onset of the VMR usually occurred within 2 s of laser stimulation as typically seen with distension, and generally continued for the duration of the stimulation, with occasional after-discharges.

**Figure 4:**
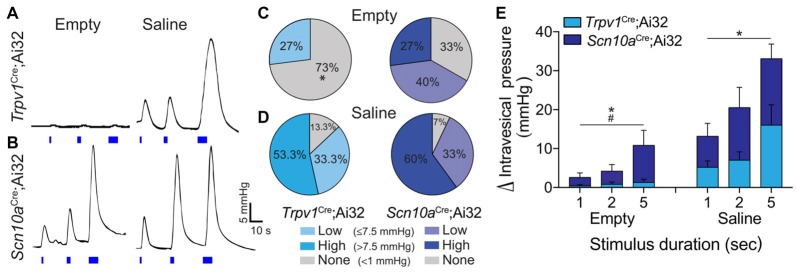
Optogenetic stimulation of ChR2+ bladder afferents revealed population-specific effects on bladder contractile activity.** (A,B)** Representative traces of ΔIVP in response to 1, 2 and 5 s light stimulation (blue bars) of peripheral bladder afferent terminals in *Trpv1*^Cre^;Ai32 and *Scn10a*^Cre^;Ai32 mice when bladders were drained prior to light stimulation (empty) and following saline distension to 80% of micturition threshold (MT; saline). **(C,D)** Graphs show the percentage of stimulation trials in *Trpv1*^Cre^;Ai32 and *Scn10a*^Cre^;Ai32 mice in which low amplitude (>1.0 but <7.5 mmHg) or high amplitude (≥7.5 mmHg) bladder contractions, or no change in intravesical pressure (IVP), were recorded in response to light stimulation during empty and saline conditions (*P < 0.05, Chi-square; *n* = 5 per group). **(E)** ΔIVP when the bladder was drained prior to light stimulation (empty) was dependent on stimulus duration (*P < 0.05) but not mouse line (*P* = 0.08), with a significant interaction (^#^*P* < 0.05). There was also an effect of stimulus duration (*P < 0.05) when the bladder was pre-distended (saline), but no effect of mouse line (*P* > 0.05) and no interaction (*P* > 0.05). Two way ANOVAs with repeated measures, *n* = 5 per group.

To determine the capacity to which optogenetic activation of peripheral afferent terminals can drive micturition reflex activity, *in vivo* bladder contractile responses to light stimulation were measured in *Trpv1*^Cre^;Ai32 and *Scn10a*^Cre^;Ai32 mice. First, IVP during saline infusion was measured to assess normal bladder function in each mouse. Saline infusion elicited low amplitude (ΔIVP < 7.5 mmHg) bladder contractile activity previously described as “non-voiding” contractions (Birder et al., 2002), that were followed by larger (ΔIVP ≥ 7.5 mmHg), phasic contractions indicative of voiding (Cockayne et al., 2000). There were no differences between *Trpv1*^Cre^;Ai32 (*n* = 5) and *Scn10a*^Cre^;Ai32 (*n* = 5) mice in baseline IVP (2.83 ± 0.76 mmHg and 2.17 ± 0.51 mmHg, respectively), nor were there differences in MT (0.14 ± 0.06 ml and 0.13 ± 0.05 ml, respectively) or PT (14.62 ± 2.26 mmHg and 17.53 ± 3.42 mmHg, respectively). In the same mice, the effects of ChR2-mediated depolarization of peripheral afferent terminals on bladder contractile activity was examined. In both mouse lines, the amplitude of light-evoked bladder contractions was duration-dependent, such that longer light pulses elicited a larger ΔIVP (Figures 4A,B,E); however, differential effects were observed between *Trpv1*^Cre^;Ai32 and *Scn10a*^Cre^;Ai32 mice. Light stimulation was more likely to produce bladder contractions in *Scn10a*^Cre^;Ai32 than in *Trpv1*^Cre^;Ai32 mice when the bladder was empty ($\chi_{\left(1\right)}^{2}$ = 4.821, *P* < 0.05). Stimulation of the empty bladder elicited low amplitude (>1.0 but <7.5 mmHg), “non-voiding” contractions in 27% (4/15) of the trials in *Trpv1*^Cre^;Ai32 mice and 40% (6/15) of the trials in *Scn10a*^Cre^;Ai32 mice (Figure 4C). Micturition contractions (>7.5 mmHg) were observed in 0% (0/15) of the empty trials in *Trpv1*^Cre^;Ai32 mice and 27% (4/15) in *Scn10a*^Cre^;Ai32 mice (Figure 4C). Because physiological micturition contractions occur as a result of bladder filling-induced activity of LT, mechanosensitive afferents, a second set of light stimulation trials was performed after saline was infused into the bladder to reach 80% of the MT, as initially determined for each mouse. In this condition, there was no difference in the likelihood of light-evoked contractions, which occurred in 93% (14/15) of the trials in *Scn10a*^Cre^;Ai32 and 87% (13/15) of the trials in *Trpv1*^Cre^;Ai32 mice ($\chi_{\left(1\right)}^{2}$ = 2.143, *P* > 0.05). The majority of contractions were ≥7.5 mmHg in both groups (Figure 4D). When two-way repeated measures ANOVAs were used to analyze the effects of stimulus duration on ΔIVP (Figure 4E), there was a significant main effect of stimulus duration regardless of bladder filling, which did not depend on the fiber types being stimulated. There was, however, a significant interaction between these two groups and stimulus duration when the bladder was empty, such that the ΔIVP was greater with longer stimulation in *Scn10a*^Cre^;Ai32 mice (*F*_(2,16)_ = 3.779, *P* < 0.05).

### Differential Spinal Synaptic Communication between *Trpv1*^Cre^;Ai32 and *Scn10a*^Cre^;Ai32 Mice

**Figure 5:**
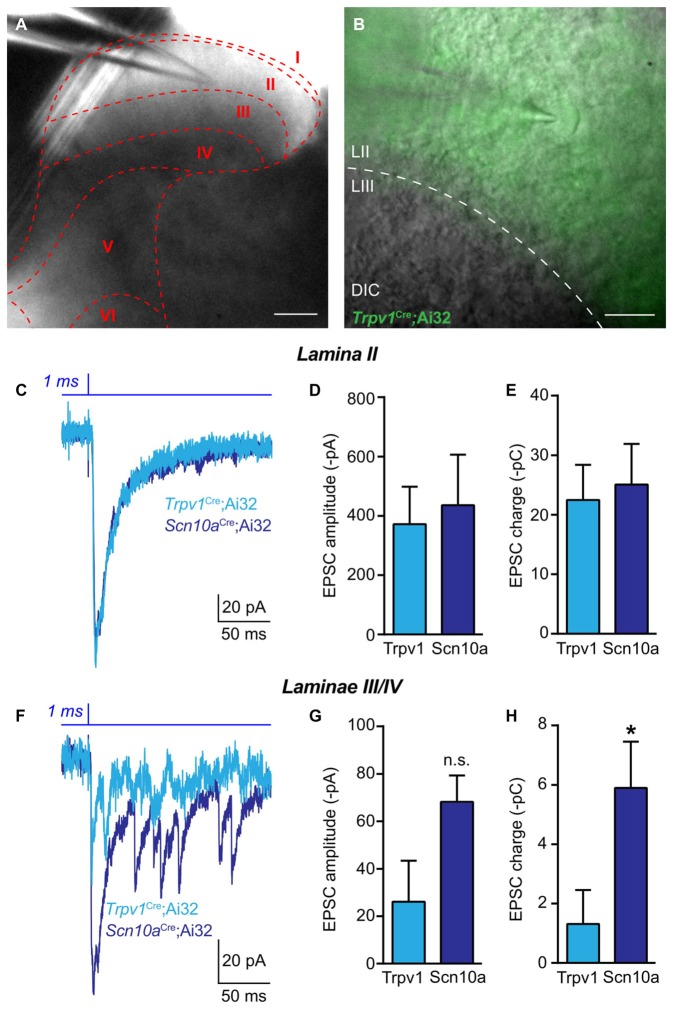
Spinal functional connectivity in *Trpv1*^Cre^;Ai32 and *Scn10a*^Cre^;Ai32 mice. **(A,B)** Representative images showing location of recording electrode in spinal cord slice from *Trpv1*^Cre^;Ai32 mice. **(C)** Representative traces recorded during optogenetic activation of excitatory synaptic transmission from ChR2+ primary afferents in voltage clamp recordings from laminae I-II in *Trpv1*^Cre^;Ai32 and *Scn10a*^Cre^;Ai32 mice. **(D,E)** In *Trpv1*^Cre^;Ai32 and *Scn10a*^Cre^;Ai32 mice, there was no significant difference in the amplitude of EPSCs or charge transfer (*P* > 0.05, unpaired *t*-test; *n* = 9 cells for *Trpv1*^Cre^;Ai32 and *n* = 5 cells for *Scn10a*^Cre^;Ai32). **(F)** Representative traces of excitatory synaptic currents during voltage clamp recordings from laminae III/IV in *Trpv1*^Cre^;Ai32 and *Scn10a*^Cre^;Ai32 mice. **(G)** There was no significant difference between in the amplitude of excitatory currents. **(H)** In spinal laminae III/IV of *Scn10a*^Cre^;Ai32 mice, a significant increase in charge transfer was observed compared to *Trpv1*^Cre^;Ai32 mice (*P < 0.05, unpaired *t*-test; *n* = 7 cells for *Trpv1*^Cre^;Ai32 and *n* = 6 cells for *Scn10a*^Cre^;Ai32).

To further examine potential divergent roles of lineage *Trpv1*+ and *Scn10a*+ afferents, we assessed differences in spinal cord synaptic connectivity in *Trpv1*^Cre^;Ai32 *and Scn10a*^Cre^;Ai32 mice. We performed whole-cell patch-clamp recordings from acute LS spinal cord slices (Figures 5A,B) and used brief (1 ms) pulses of 470 nm light to stimulate ChR2-expressing primary afferent central terminals (Figure 5B). Recordings from neurons in laminae I-II, which exhibited similar patterns of eYFP expression in both mouse lines (Figures 1I,J), revealed no difference in amplitude of excitatory postsynaptic currents (EPSCs; Unpaired *t*-test; *n* = 5–9 cells; Figures 5D,E). We also calculated the charge transfer to quantify polysynaptic input onto neurons, and found no difference between these transgenic lines in laminae I/II (Figure 5C). However, in recordings from neurons in deeper laminae (III/IV) that are more densely innervated by ChR2+ afferents in *Scn10a*^Cre^;Ai32 mice, we found enhanced EPSC amplitudes and significantly increased charge transfer compared to deeper laminae recordings in *Trpv1*^Cre^;Ai32 mice (Unpaired *t*-test, *P* < 0.05; *n* = 6–7 cells; Figures 5F–H).

## Discussion

In the current study, we demonstrate successful application of optogenetics to *in vivo* modulation of bladder sensation as it relates to both nociception and voiding. A transgenic strategy was used to express ChR2 in select populations of afferents via Trpv1^Cre^ (Cavanaugh et al., 2011b) and SNS^Cre^ (i.e., *Scn10a*^Cre^; Agarwal et al., 2004). Photostimulation of ChR2+ peripheral bladder afferent terminals differentially modulated bladder sensory pathways in *Trpv1*^Cre^;Ai32 and *Scn10a*^Cre^;Ai32 mice. Light stimulation significantly enhanced nociceptive reflex behavior (VMR) evoked by a range of noxious distension pressures (30–50 mmHg) in both ChR2-expressing mouse lines. Further, stimulation in *Scn10a*^Cre^;Ai32, but not *Trpv1*^Cre^;Ai32, mice potentiated the VMR to non-noxious bladder distension, suggesting that alterations in the threshold or functional activity of this population may be important for sensations of discomfort or urgency at physiologic IVPs.

In voiding reflex studies, contractile activity of the bladder was elicited by peripheral afferent terminal activation that depended on the fiber types targeted. Afferent depolarization in *Trpv1*^Cre^;Ai32 mice elicited little or no contractile activity unless the bladder was partially filled with saline. This prerequisite engagement of low-threshold mechanosensitive afferents is in agreement with the longstanding hypothesis that C-fiber afferents primarily act to modulate, rather than initiate, micturition. In contrast, photostimulation of bladder afferents in *Scn10a*^Cre^;Ai32 mice elicited both small (<7.5 mmHg) and large (≥7.5 mmHg) amplitude contractile activity whether the bladder was empty or partially filled. This finding is consistent with evidence that bladder afferents terminating in deeper laminae directly engage micturition pathways. In *Scn10a*^Cre^;Ai32 mice, eYFP+ fibers projected densely throughout laminae I-V, into lamina X, and throughout the dorsal columns. However, it is worth noting that eYFP+ fibers did not appear to terminate on parasympathetic preganglionic neurons in the SPN region of the spinal cord; rather, they terminate on interneurons that synapse on these preganglionic neurons or ascend to engage the bulbo-spinal micturition pathway. An additional possibility is that connectivity between central ChR2+ afferent projections and the lateral spinal nucleus (LSN) is enhanced in *Scn10a*^Cre^;Ai32 compared to *Trpv1*^Cre^;Ai32 mice. The LSN has a demonstrated role in polysynaptic transmission and/or modulation of afferent sensory information with preference for deep tissues, and in fact receives sparse, but direct, visceral afferent input (Sikandar et al., 2017). In turn, the LSN projects to and receives projections from a large number of supraspinal sites important for sensory processing and modulation of both pain and micturition (e.g., periaqueductal gray, thalamus, hypothalamus, amygdala; Burstein et al., 1987; Harmann et al., 1988; Battaglia and Rustioni, 1992; Burstein and Potrebic, 1993). Thus, although the LSN may contribute to enhancement of distension-evoked nociceptive responses by light stimulation in both *Scn10a*^Cre^;Ai32 and *Trpv1*^Cre^;Ai32 mice, it may also contribute to differential contractile activity depending on the identity of the stimulated fibers through additional mechanisms discussed below.

There are several additional potential explanations for differences in photostimulation effects, including the fiber types of ChR2+ terminals (myelinated vs. unmyelinated), the response threshold (LT vs. HT) of ChR2+ bladder afferents, and/or the total number of bladder-innervating ChR2+ neurons in *Trpv1*^Cre^;Ai32 and *Scn10a*^Cre^;Ai32 mouse lines. In contrast to somatic nerves in which CTβ is transported by neurons with myelinated axons and conduction velocities in the A-fiber range (Robertson and Grant, 1989; LaMotte et al., 1991), CTβ labels a combination of myelinated and unmyelinated visceral afferent neurons (Wang et al., 1998; Fasanella et al., 2008; Zhong et al., 2008). Thus, it is possible that *Scn10a*^Cre^;Ai32 and *Trpv1*^Cre^;Ai32 mice express differing proportions of ChR2-eYFP+ neurons with myelinated vs. unmyelinated fibers. Using immunohistochemical techniques, TRPV1 has been identified as a neurochemical marker preferentially expressed by unmyelinated visceral afferents in rat (De Schepper et al., 2008; Forrest et al., 2013) and in mouse (Fasanella et al., 2008; Zhong et al., 2008). In support of this, previous work on mouse colon afferents found that 87% were TRPV1-positive, all of which had conduction velocities in the range of C-fiber afferents, and had high response thresholds to distension with low firing frequency that remained relatively constant across stimulus intensities (Malin et al., 2009). This is in contrast to neurochemical studies suggesting that targeting Na_v_1.8^+^ neurons includes the majority of C-fibers, including peptidergic and non-peptidergic nociceptors, VGLUT3+ low-threshold mechanosensitive types, and in nearly one-third of NF200+, A-fiber afferents (Black et al., 1996, 2003; Shields et al., 2012). The majority of bladder afferent neurons in rat exhibit TTX-resistant Na^+^ currents that contribute to high electrical thresholds for action potential firing (Yoshimura et al., 1996). This population is significantly reduced by capsaicin treatment, suggesting that a significant proportion of these afferents express TRPV1 (Yoshimura et al., 1996). Experimental reduction in the expression of Na_v_1.8 via antisense oligodeoxynucleotide treatment reduces TTX-resistant Na^+^ conductance in bladder afferent neurons, indicating that the aforementioned population overlap with Na_v_1.8-expressing neurons (Yoshimura et al., 2001). Thus, one might reasonably extrapolate that ChR2-expressing bladder afferents in Trpv1^Cre^;Ai32 mice in the current study comprise the high threshold, TTX-resistant (Na_v_1.8^+^) population of C-fibers that do not normally respond to physiological bladder filling. This notion is supported by the prerequisite engagement of low threshold mechanosensitive afferents by sub-threshold saline infusion to generate large amplitude bladder contractions in the cystometric studies reported here. In turn, the higher percentage (by 20%–30%) of ChR2-expressing bladder afferents in Scn10a^Cre^;Ai32 mice (relative to the Trpv1^Cre^; Ai32 mice) likely represents the low-threshold population of afferents that normally respond to bladder filling.

Previous characterization of *Trpv1*^Cre^ mice demonstrated that this transgenic line targets both peptidergic and non-peptidergic C-fibers (Cavanaugh et al., 2011a). This is confirmed by eYFP+ central projections in *Trpv1*^Cre^;Ai32 fibers that were largely restricted to the superficial DH, i.e., laminae I-II, where the majority of peptidergic and non-peptidergic C-fiber afferents respectively terminate. Thus, the appearance of eYFP+ projections to laminae III-V and heavier projections to lamina X in *Scn10a*^Cre^;Ai32 mice is likely due to the expression of ChR2-eYFP in myelinated A-fiber afferents in addition to C-fiber afferents (Shields et al., 2012). In spinal cord slice electrophysiology experiments, we observed elevated functional connectivity in *Scn10a*^Cre^;Ai32 mice between ChR2+ afferents and second order neurons in deeper (III-IV), but not superficial (I-II), laminae of the DH compared to ChR2+ fibers in *Trpv1*^Cre^;Ai32 mice. Although the vast majority of bladder afferent input to the DH is to laminae I, V–VII and X, sparse inputs to laminae III-IV occasionally have been shown by pseudorabies virus labeling or c-Fos expression (e.g., Vizzard et al., 1996; Vizzard, 2000). Speculatively, it is possible that Aδ-fibers labeled by ChR2-eYFP in *Scn10a*^Cre^;Ai32 mice contributes to the enhanced synaptic communication between incoming afferents and the neurons located in deeper laminae, resulting in differential processing of visceral information conveyed by sensory neurons innervating the bladder. That excitation of myelinated, Aδ-fiber afferents in *Scn10a*^Cre^;Ai32 mice may be responsible for driving the voiding contractions is also in accordance with previous rat studies that have utilized chemical desensitization of capsaicin-sensitive bladder afferents (i.e., TRPV1+ C-fibers) to show that Aδ-fiber afferents mediate normal micturition (Cheng et al., 1993).

Alternatively, increased visceromotor and voiding reflex behavior in *Scn10a*^Cre^;Ai32 mice could be due to stimulation of a greater overall number of ChR2+ bladder afferents than in *Trpv1*^Cre^;Ai32 mice. Compared to 62% in *Trpv1*^Cre^;Ai32 mice, 82.2% of retrogradely labeled LS DRG neurons expressed ChR2-eYFP in *Scn10a*^Cre^;Ai32 mice. Previous reports (La et al., 2011; DeBerry et al., 2014) of *Trpv1* mRNA and functional protein expression in ~60% of LS bladder afferents are in accordance with the present results. The observed proportion of eYFP+ bladder afferents in *Scn10a*^Cre^;Ai32 mice was slightly higher than previous reports of Na_v_1.8 expression in 75% of mouse DRG neurons (Shields et al., 2012), and higher than the 70%–74% of bladder afferents in rat that exhibit TTX-resistant, Na_v_1.8-mediated sodium currents (Yoshimura et al., 1996, 2006; Masuda et al., 2006). It is important to note that in all of these studies and in the present study, retrograde labeling of afferent somata was achieved by microinjection of CTβ into the bladder parenchyma, which labels a distinct anatomical subset of non-urothelial, bladder-innervating primary afferents (Kanda et al., 2016; Clodfelder-Miller et al., 2017). Whether there are differences in the proportion of peri-urothelial afferents in *Trpv1*^Cre^;Ai32 vs. *Scn10a*^Cre^;Ai32 mice and whether peri-urothelial afferents provide sensory input that is unique from that of the non-urothelial afferents studied here remains to be determined.

Expression of ChR2 in the CNS has been used to study a number of behaviors, including those related to anxiety, learning, and depression (Huber et al., 2008; Covington et al., 2010; Tye et al., 2011). Recently, optogenetic modulation of bladder smooth muscle was shown to elicit voiding *in vivo* (Park et al., 2017), and our group demonstrated that inhibition of sensory afferent activity via archaerhodopsin, a light-activated proton pump, attenuates pain-related behavior in the context of bladder inflammation (Samineni et al., 2017a). The present study is an important addition that demonstrates for the first time the effects of direct, selective neuronal activation of distinct bladder afferents on behavioral correlates of bladder sensation and function. Selective optogenetic activation of primary afferent activity differentially modulated transmission of nociceptive sensory information and autonomic reflexes initiated by primary sensory afferents. This optogenetic approach may also prove useful for understanding pain processing in other visceral systems given that sensory afferents, including those that innervate the parenchyma and vasculature of a given organ, are important for both pain and homeostasis.

## Ethics Statement

All experiments were conducted in accordance with the National Institute of Health guidelines and with approval from the Institutional Animal Care and Use Committees of University of Alabama at Birmingham, University of Pittsburgh, and Washington University School of Medicine.

## Author Contributions

JJD and VKS collected the data. JJD, VKS, BMD and RWG designed the experiments, analyzed the data and wrote the manuscript. BAC performed electrophysiological experiments. SKV and CJS performed breeding and anatomical analyses. KMA provided resources.

## Conflict of Interest Statement

The authors declare that the research was conducted in the absence of any commercial or financial relationships that could be construed as a potential conflict of interest.
